# Viral suppression is associated with rising NT-proCNP levels and reduced systemic inflammation in people living with HIV

**DOI:** 10.1007/s15010-026-02812-z

**Published:** 2026-05-11

**Authors:** Istvan Bojti, Sarolta Bojtine Kovacs, Antonia Ziegler, Noah Jung, Alexander Maier, Dirk Westermann, Jean-Luca Funk, Philipp Mathé, Geertje Fink, Susanne Usadel, Siegbert Rieg, Gabor Tamas Szabo, Daniel Czuriga, Matthias C. Müller

**Affiliations:** 1 Department of Cardiology and Angiology, University Heart Center Freiburg, Faculty of Medicine, University of Freiburg, Freiburg, Germany; 2 IMM-PACT Cluster of Excellence, Faculty of Medicine, University of Freiburg, Freiburg, Germany; 3 Section of Molecular Hematology, Department of Medicine I, Hematology, Oncology and Stem Cell Transplantation, Medical Center-University of Freiburg, Faculty of Medicine, University of Freiburg, Freiburg, Germany; 4 Division of Infectious Diseases, Department of Medicine II, Medical Center-University of Freiburg, Faculty of Medicine, University of Freiburg, Freiburg, Germany; 5 Department of Infection Medicine, Medical Service Centre Clotten, Freiburg, Germany; 6 Division of Cardiology, Department of Cardiology, Faculty of Medicine, University of Debrecen, Debrecen, Hungary

**Keywords:** HIV, NT-proCNP, Inflammation, ART

## Abstract

**Background:**

People living with HIV (PLWH) have an increased risk of cardiovascular disease that persists despite effective antiretroviral therapy (ART) and viral suppression. Chronic inflammation and endothelial dysfunction are central contributors, yet integrative biomarkers linking viral activity, inflammation, and vascular homeostasis remain poorly defined. N-terminal pro-C-type natriuretic peptide (NT-proCNP), a marker of endothelial function and cardiovascular stress, is responsive to inflammatory states, and is processed by furin, a protease also required for viral entry. Data on NT-proCNP in PLWH are scarce. The aim of this study was to investigate longitudinal changes in NT-proCNP in relation to HIV viral load and systemic inflammation in PLWH starting ART.

**Methods:**

In this exploratory longitudinal observational study, we included PLWH aged ≥ 18 years with initial diagnosis of HIV between 09/2023 and 06/2025. Before and approximately 3 months after initiation of ART plasma NT-proCNP, furin, and a multiplex panel of inflammatory cytokines were measured alongside HIV viral load, CD4⁺ T-cell count, serum creatinine, and C-reactive protein. Paired comparisons were performed using the Wilcoxon matched-pairs signed-rank test with false discovery rate (FDR) correction using the Benjamini–Krieger–Yekutieli method (Q = 5%). Correlations between longitudinal changes were explored using Spearman analysis with FDR adjustment.

**Results:**

NT-proCNP concentrations increased significantly over time (33.7 [25.3–40.7] vs. 39.4 [31.2–55], *p* = 0.006), while a broad range of inflammatory cytokines showed significant reduction after FDR correction. Circulating furin concentrations did not change significantly. Correlation analyses between changes in NT-proCNP and individual inflammatory or virological markers were not significant after FDR correction.

**Conclusion:**

In this study we present first-time data on the impact of the initiation of ART on the levels and dynamics of NT-proCNP in PLWH. Suppression of HIV replication is accompanied by reduced systemic inflammation and a significant increase in circulating NT-proCNP, suggesting the restoration of vascular homeostatic signaling during early follow-up. NT-proCNP may represent an integrative biomarker linking viral control, inflammation, and cardiovascular biology in HIV infection, warranting further investigation in larger prospective studies with clinical cardiovascular endpoints.

**Trial registration number and date of registration:**

NCT02149004, 23.05.2014.

**Supplementary Information:**

The online version contains supplementary material available at 10.1007/s15010-026-02812-z.

## Introduction

People living with HIV (PLWH) experience a substantially increased risk of cardiovascular (CV) morbidity and mortality compared with the general population, even in the era of effective antiretroviral therapy (ART) [1]. This excess risk cannot be fully explained by traditional CV risk factors alone and is thought to arise from a complex interplay between persistent low-level HIV replication, latent co-infections such as CMV, immune activation, systemic inflammation, and endothelial dysfunction [2]. Identifying biomarkers that integrate viral activity, inflammatory burden, and cardiovascular homeostasis remains a major unmet need in HIV research [3].

C-type natriuretic peptide (CNP) is a key regulator of vascular homeostasis, exerting vasodilatory, anti-inflammatory, and anti-fibrotic effects [4]. Due to its short circulating half-life, the stable precursor fragment N-terminal proCNP (NT-proCNP) is commonly used as a surrogate marker of CNP production. NT-proCNP has emerged as a sensitive indicator of endothelial function as well as cardiovascular stress and has been associated with inflammatory states and adverse cardiovascular outcomes [5–7]. Given the central role of endothelial dysfunction in HIV-associated cardiovascular disease, NT-proCNP represents a biologically plausible candidate biomarker in this population.

A mechanistic link between viral infections and NT-proCNP regulation may involve the proprotein convertase furin. Furin is responsible for intracellular processing of numerous host and viral proteins, including the maturation of CNP [8]. Importantly, furin is also required for the activation of viral envelope glycoproteins, facilitating cellular entry and infectivity of several viruses, including HIV [9]. This shared dependence on furin suggests that viral replication and CNP processing may be interconnected at a cellular level. However, the direct assessment of biologically relevant furin activity in humans remains challenging, as circulating furin concentrations correlate poorly with intracellular enzymatic activity [10].

Recent evidence supports the concept of a virus-CNP axis. In patients with COVID-19, NT-proCNP levels were shown to correlate with viral load and disease severity, suggesting that CNP may reflect both viral burden and host response [11]. HIV infection, characterized by long-term viral persistence and chronic immune activation, represents a particularly suitable model to investigate this relationship. NT-proCNP has also been described as an inflammatory marker, and its relationship with systemic inflammation in HIV remains incompletely understood [12].

The present exploratory study therefore aimed to investigate longitudinal changes in NT-proCNP in relation to HIV viral load during early follow-up in PLWH starting ART. In addition, we assessed the association between NT-proCNP and a broad panel of inflammatory markers, alongside furin concentrations, to explore their potential interrelationships in PLWH.

## Methods

### Study design and population

This exploratory longitudinal observational study included PLWH aged ≥ 18 years with first diagnosis of HIV between 09/2023 and 06/2025. All PLWH were recruited in an outpatient care setting in Freiburg, Germany and provided written informed consent. Eligibility required availability of cryopreserved paired plasma samples and HIV viral load measurements at baseline (T0) before starting ART and at a follow-up visit approximately three months after starting ART (T1). Exclusion criteria included acute intercurrent infections at sampling and conditions known to substantially influence natriuretic peptide metabolism, such as advanced renal disease (Serum creatinine above 1.5 mg/dl at screening). The study was conducted in accordance with the Declaration of Helsinki and approved by the local ethics committee. Written informed consent was obtained from all participants.

### Sample collection and processing

Peripheral venous blood was collected into EDTA tubes and processed within two hours of collection. Plasma was separated by centrifugation, aliquoted, and stored at − 80 °C until analysis. Repeated freeze–thaw cycles were avoided.

### HIV viral load and routine laboratory parameters

Plasma HIV-1 RNA levels were quantified using a clinically validated nucleic acid amplification assay and reported as copies/mL. Additional routine laboratory parameters included CD4⁺ T-cell count, serum creatinine, and C-reactive protein (CRP), measured using standard clinical laboratory methods.

### NT-proCNP measurement

NT-proCNP concentrations were quantified using a commercially available sandwich ELISA (Biomedica NT-proCNP ELISA, Biomedica Medizinprodukte GmbH, Vienna, Austria) according to the manufacturer’s instructions. Samples were measured in duplicate, and mean values were used for analysis. Quality control samples and standard curves were included on each plate.

### Furin measurement

Plasma furin concentrations were measured using a sandwich ELISA (ABIN6966912, antibodies-online GmbH). Measurements were performed in duplicate. Given the predominantly intracellular activity of furin and the limited correlation between circulating levels and enzymatic function, furin analyses were considered exploratory.

### Inflammatory biomarker assessment

Inflammatory cytokines and chemokines were measured using a multiplex bead-based immunoassay (LEGENDplex™ Human Inflammation Panel 1, 13-plex; BioLegend). Data acquisition was performed by flow cytometry, and concentrations were calculated using manufacturer-provided standards and software.

### Statistical analysis

This study was exploratory in nature. Continuous variables are presented as median with interquartile range (IQR). Paired comparisons between baseline (T0) and the 3-month follow-up visit (T1) were performed using the Wilcoxon matched-pairs signed-rank test, which compares the ranks of paired observations and does not assume normal data distribution. To account for multiple comparisons, false discovery rate (FDR) correction was applied using the two-stage step-up procedure of Benjamini, Krieger, and Yekutieli, with a desired FDR (Q) of 5%. All p values reported in the Results section are FDR-adjusted and were interpreted in a hypothesis-generating context. Correlations between longitudinal changes in NT-proCNP and changes in virological, immunological, and inflammatory parameters were explored using Spearman rank correlation analysis based on delta values (Δ = 3-month follow-up minus baseline). Resulting p values from these correlation analyses were adjusted for multiple testing using false discovery rate (FDR) correction with the two-stage Benjamini–Krieger–Yekutieli procedure (Q = 5%), and all reported p values represent FDR-adjusted q values. All statistical tests were two-sided. No multivariable modeling was performed. Statistical analyses were conducted using GraphPad Prism version 10.4.2 (GraphPad Software, San Diego, CA, USA).

## Results

A total of 23 PLWH who met all predefined inclusion and exclusion criteria were enrolled in the study, and all subsequent analyses were conducted in this final cohort [(age median: 39.8 years; IQR 27.5–58.4), (sex: 87% male)]. The median time between start of ART and T1 was 109 days (IQR 92–113). With regard to ART, the majority of participants received integrase strand transfer inhibitor (INSTI)–based regimens (*n* = 18), including bictegravir/emtricitabine/tenofovir alafenamide (*n* = 13), dolutegravir/lamivudine (*n* = 3), and dolutegravir/emtricitabine/tenofovir (*n* = 2). Non-nucleoside reverse transcriptase inhibitor (NNRTI)–based therapy with doravirine/lamivudine/tenofovir disoproxil was used in two participants (*n* = 2), while three participants received blinded study medication (*n* = 3).

### HIV viral load and immunological parameters

**Fig. 1:**
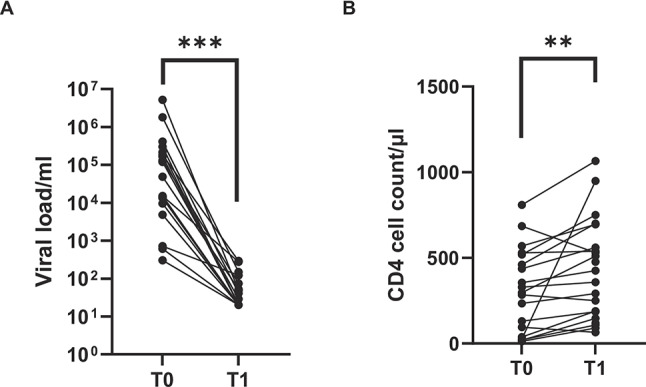
Virological suppression and immunological recovery during follow-up. Paired changes in **A** HIV viral load and **B** CD4⁺ T-cell count between baseline (T0) before starting ART and follow-up visit (T1) 3-months after initiation of ART. Each line represents an individual participant. Data are shown as paired dot plots. Viral load is displayed on a logarithmic scale. Paired comparisons were performed using the Wilcoxon matched-pairs signed-rank test. P values were adjusted for multiple comparisons using false discovery rate (FDR) correction with the two-stage Benjamini–Krieger–Yekutieli procedure (Q = 5%). Asterisks indicate FDR-adjusted p values (q values) as follows: *q < 0.05; **q < 0.01; ***q < 0.001

HIV viral load decreased markedly between T0 and T1(83700 [8485-237174] vs. 24.5 [20–62], p = < 0.001)). CD4⁺ T-cell counts increased significantly over the same period (297 [66–524] vs. 468 [175–696], *p* = 0.002)) (Fig. 1).

### NT-proCNP and furin

**Fig. 2:**
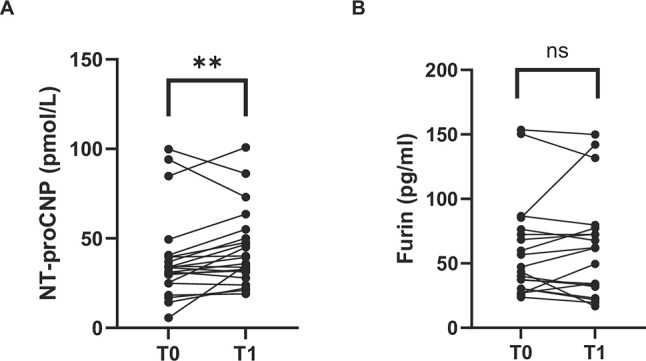
Longitudinal changes in NT-proCNP and circulating furin concentrations. Paired plasma concentrations of **A** NT-proCNP and **B** furin at baseline (T0) before starting antiretroviral therapy (ART) and follow-up visit (T1) 3-months after initiation of ART. Individual participant trajectories are shown as connected data points. Paired comparisons were performed using the Wilcoxon matched-pairs signed-rank test with false discovery rate (FDR) correction applied using the Benjamini–Krieger–Yekutieli method (Q = 5%). Asterisks denote FDR-adjusted p values (q values): *q < 0.05; **q < 0.01; ***q < 0.001. “ns” indicates not significant after FDR correction

NT-proCNP concentrations increased significantly from baseline to follow-up (33.7 [25.3–40.7] vs. 39.4 [31.2–55], *p* = 0.006), with the majority of individuals showing higher levels at T1 (74%). Plasma furin concentrations did not differ significantly between baseline and follow-up (45.3 [29.5–78.6] vs. 64.9 [32.2–85.7], *p* = 0.097) (Fig. 2).

### Inflammatory biomarkers

**Fig. 3:**
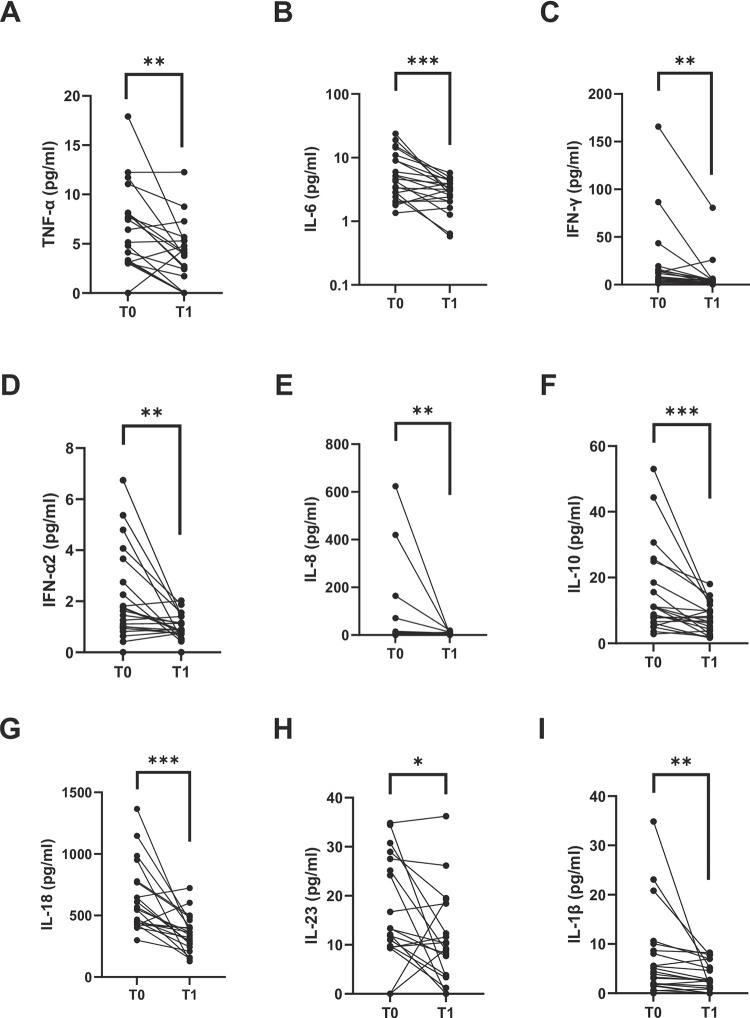
Reduction of systemic inflammatory markers during follow-up. Paired plasma concentrations of inflammatory cytokines that showed a significant change between baseline (T0) before starting ART and follow-up visit (T1) 3-months after initiation of ART. False discovery rate (FDR) correction are shown: **A** TNF-α, **B** IL-6, **C** IFN-γ, **D** IFN-α2, **E** IL-8, **F** IL-10, **G** IL-18, **H** IL-23 and **I** IL-1β. Individual participant values are displayed as paired dot plots. Paired comparisons were performed using the Wilcoxon matched-pairs signed-rank test. P values were adjusted for multiple testing using false discovery rate (FDR) correction with the two-stage Benjamini–Krieger–Yekutieli procedure (Q = 5%). Asterisks indicate FDR-adjusted p values (q values) as follows: *q < 0.05; **q < 0.01; ***q < 0.001

A broad reduction in systemic inflammatory markers was observed. TNF-α (4.8 [3.11–8.06] vs. 3.8 [0–5.3], *p* = 0.006), IL-6 (4.6 [2.4–11] vs. 3 [2.1–3.9], p = < 0.001), IFN-γ (8.1 [3.3–14.7] vs. 4.1 [1.6–5.3], *p* = 0.006), IFN-α2 (1.5 [1–2.8] vs. 0.9 [0.7–1.4], *p* = 0.004), IL-8 (8.5 [0–15.7] vs. 4.7 [1.4–6.5], *p* = 0.005), IL-10 (10.8 [6.1–18.5] vs. 6.8 [3.8–9.9], p = < 0.001), IL-18 (554.1 [439.8–778.2] vs. 365.7 [269.4–487.1], p = < 0.001), IL-23 (12.3 [9.4–25.1] vs. 9.2 [3.3–18.4], *p* = 0.027), and IL-1β (3.88 [1.6–8.6] vs. 2.7 [1.01–5.2], *p* = 0.002) all decreased significantly between T0 and T1. These changes indicate attenuation of immune activation during follow-up (Fig. 3).

### CRP and renal function

**Fig. 4:**
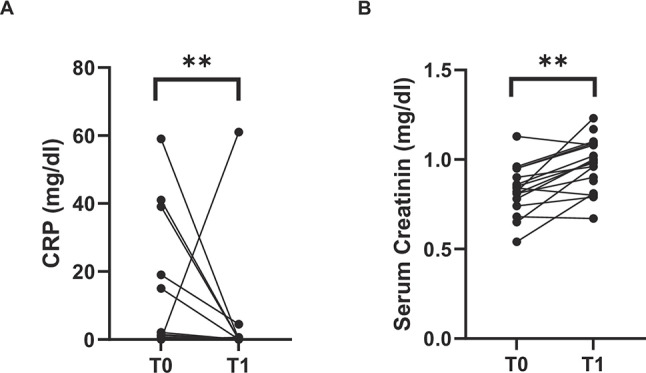
Renal function and systemic inflammation markers. Paired measurements of **A** C-reactive protein (CRP) and **B** serum creatinine at baseline (T0) before starting ART and follow-up visit (T1) 3-months after initiation of ART. Individual participant data are shown. Paired comparisons were performed using the Wilcoxon matched-pairs signed-rank test with false discovery rate (FDR) correction (Benjamini–Krieger–Yekutieli, Q = 5%). Asterisks denote FDR-adjusted p values (q values): *q < 0.05; **q < 0.01; ***q < 0.001

 CRP-levels showed a significant decrease (0.6 [0–2.1] vs. 0 [0–0], *p* = 0.006). Serum creatinine increased modestly between T0 and T1 (0.84 [0.76–0.95] vs. 0.99 [0.8–1.1], *p* = 0.005) No individual exhibited an increase greater than 1.5-fold from baseline or an absolute value exceeding 1.3 mg/dL.(Fig. 4).

### Correlation analysis

Changes in NT-proCNP were explored in relation to longitudinal changes in virological, immunological, and inflammatory parameters using Spearman correlation analysis. Although several correlations showed nominal associations at the unadjusted level, none of the correlations remained statistically significant after false discovery rate (FDR) correction (q > 0.05 for all comparisons).

## Discussion

In this exploratory longitudinal study, we present for the first time data showing the effect of the initiation of ART on NT-proCNP levels in PLWH. Suppressing HIV replication was accompanied by a significant increase in circulating NT-proCNP levels and a broad reduction in systemic inflammatory mediators. These findings support the hypothesis that NT-proCNP may reflect changes in vascular homeostasis associated with viral control and resolution of inflammation in PLWH starting ART.

Chronic immune activation and inflammation are central drivers of cardiovascular disease in HIV infection [13]. Even under effective ART, residual inflammasome-driven inflammation persists and contributes to vascular pathology. Notably, inhibition of IL-1β has been shown to reduce atherosclerotic arterial inflammation in treated HIV, underscoring the clinical relevance of inflammatory pathways beyond virological suppression [14]. In this context, the observed increase in NT-proCNP with viral suppression and declining systemic inflammation may reflect the restoration of vascular homeostatic signaling rather than the direct coupling to individual inflammatory mediators, consistent with the role of CNP as an integrative regulator of endothelial function [15].

Beyond its vascular effects, CNP has also been implicated in the regulation of platelet reactivity and thrombosis. Experimental data indicate that CNP inhibits platelet activation and aggregation via cyclic GMP–dependent pathways, linking endothelial CNP signaling to antithrombotic homeostasis [16]. This is of particular relevance in HIV infection, where persistent immune activation and endothelial dysfunction are associated with increased platelet reactivity and a prothrombotic state, even under effective ART [17]. Thus, changes in NT-proCNP levels during viral suppression may also reflect modulation of endothelial–platelet interactions that contribute to thrombotic and cardiovascular risk in PLWH.

Despite the known involvement of furin in both viral entry and proCNP processing, circulating furin concentrations did not change significantly during follow-up. This finding is consistent with the predominantly intracellular activity of furin and the limited interpretability of serum measurements [10]. However, our results strengthen the hypothesis of decreased furin availability in the setting of viral infection, which consequently leads to not only lower NT-proCNP levels, but may also result in dysfunction of ion channels playing an important role in electrolyte and fluid homeostasis [18].

A modest increase in serum creatinine was observed; however, this remained clinically insignificant. No participant exceeded a 1.5-fold increase from baseline or an absolute creatinine concentration of 1.3 mg/dL. These findings are consistent with non-pathological changes commonly observed after initiation or modification of ART, particularly INSTI-based regimens and likely reflecting altered tubular creatinine handling rather than true renal impairment [19, 20].

Despite biologically plausible links between NT-proCNP and inflammatory pathways, no statistically significant correlations were observed after correction for multiple testing. This likely reflects the exploratory nature of the study, the modest sample size, and the conservative false discovery rate approach applied. These findings suggest that changes in NT-proCNP may not be directly proportional to short-term fluctuations in individual inflammatory mediators but rather reflect a more integrated or delayed vascular response to viral suppression.

The exploratory design and limited sample size preclude causal inference, and results should be considered hypothesis-generating. Nonetheless, these data provide a strong rationale for larger, prospective studies evaluating NT-proCNP as a biomarker of cardiovascular risk in HIV.

## Conclusion

In this exploratory longitudinal study, we showed for the first time that the suppression of HIV replication may lead to a significant increase in circulating NT-proCNP levels accompanied by the reduction of systemic inflammation. The parallel dynamics of viral suppression, inflammatory attenuation, and rising NT-proCNP suggests a potential link between NT-proCNP and biological processes relevant to vascular homeostasis in people living with HIV. Larger prospective studies are needed to determine the clinical relevance of NT-proCNP in HIV-associated cardiovascular risk.

## Supplementary Information

Below is the link to the electronic supplementary material.

Supplementary file 1

## Data Availability

The datasets generated and/or analyzed during the current study are not publicly available due to ethical and data protection restrictions related to patient confidentiality. De-identified data may be made available from the corresponding author upon reasonable request and subject to approval by the local ethics committee.
