# Supplementary material for: Viral suppression is associated with rising NT-proCNP levels and reduced systemic inflammation in people living with HIV
Source: Infection. 2026 May 11;54(4):1945–51. doi: 10.1007/s15010-026-02812-z (PMC13476305; doi:10.1007/s15010-026-02812-z)
Supplement: Supplementary file 1 [file 15010_2026_2812_MOESM1_ESM.docx]

Supplementary tables

| Supplementary table 1: Baseline characterstics | |
| --- | --- |
| n | 23 |
| Age, years (IQR) | 39.8 (27.5-58.4) |
| Sex, male (%) | 20 (87) |
| CDC classification, n (%) | |
| A1 | 4 (17) |
| A2 | 4 (17) |
| A3 | 3 (13) |
| B1 | 1 (4) |
| B2 | 2 (8) |
| B3 | 1 (4) |
| C3 | 4 (17) |
| N.A. | 4 (17) |

| **Supplementary table 2**: Absolute concentrations of the measured parameters at baseline (T0) before starting ART and follow-up visit (T1) 3-months after initiation of ART. Paired comparisons were performed using the Wilcoxon matched-pairs signed-rank test. P values were adjusted for multiple testing using false discovery rate (FDR) correction with the two-stage Benjamini–Krieger–Yekutieli procedure (Q = 5%). | | | |
| --- | --- | --- | --- |
|  | T0 | T1 | q-value |
| Viral load (cp/ml) | 83700 [8485-237174] | 24,5 [20-62] | <0,001 |
| CD4-cells (/µl) | 297 [66,5-524] | 468 [175,5-696,5] | 0,002 |
| CNP (pmol/l) | 33,7 [25,3-40,7] | 39,4 [31,2-55] | 0,006 |
| Furin (pg/ml) | 45,3 [29,5-78,6] | 64,9 [32,2-85,7] | 0,097 |
| TNF-α (pg/ml) | 4,8 [3,1-8,1] | 3,8 [0-5,3] | 0,006 |
| IL-6 (pg/ml) | 4,6 [2,4-11] | 3,0 [2,1-3,9] | <0,001 |
| IFN-γ (pg/ml) | 8,1 [3,3-14,7] | 4,1 [1,6-5,3] | 0,006 |
| IFN-α2 (pg/ml) | 1,5 [1-2,8] | 0,9 [0,7-1,4] | 0,004 |
| IL-8 (pg/ml) | 8,5 [0-15,7] | 4,8 [1,4-6,5] | 0,005 |
| IL-10 (pg/ml) | 10,8 [6,1-18,5] | 6,9 [3,8-10] | <0,001 |
| IL-18 (pg/ml) | 554,1 [439,8-778,2] | 365,7 [269,4-487,1] | <0,001 |
| IL-23 (pg/ml) | 12,3 [9,4-25,2] | 9,2 [3,3-18,4] | 0,027 |
| IL-1β (pg/ml) | 3,9 [1,6-8,6] | 2,7 [1,0-5,2] | 0,002 |
| CRP (mg/dl) | 0,6 [0-2,1] | 0 [0-0] | 0,006 |
| Creatinin (mg/dl) | 0,8 [0,8-1] | 1 [0,8-1,1] | 0,005 |
